# Relationship Between Darkness and Healing of Night Sky in Planetarium

**DOI:** 10.3390/ijerph22040569

**Published:** 2025-04-05

**Authors:** Midori Tanaka, Kenichi Otani, Takahiko Horiuchi

**Affiliations:** 1 Graduate School of Informatics, Chiba University, Yayoi-cho 1-33, Inage-ku, Chiba 263-8522, Japan; horiuchi@faculty.chiba-u.jp; 2 Konica Minolta Planetarium Co., Ltd., 3-1-3 Higashi-Ikebukuro, Toshima, Tokyo 170-8630, Japan; kenichi.otani@konicaminolta.com

**Keywords:** planetarium, starry sky, relax, light pollution

## Abstract

This study aimed to address the unexplored relaxing effects of stargazing with different night sky darknesses in a planetarium by conducting an experiment to investigate the physiological effects of the relaxation/stress state on brain and autonomic nervous system activity, as well as the psychological healing effects. Five healthy young women participated in our experiment. We conducted physiological measurements of oxygenated hemoglobin (OxyHb) concentration in the left and right prefrontal cortices by near-infrared spectroscopy, heart rate variability as a measure of the relaxation/stress state, and a psychological assessment of healing on an 11-point Likert scale. We used 9 types of stimuli: 6-star image stimuli that imitated dark star fields (low light pollution) to bright night sky (high light pollution), and movie stimuli that were viewed daily. The results showed that (1) visual stimulation with images of dark night sky significantly reduced the concentration of OxyHb in the right prefrontal cortex and (2) the psychological rating of healing was significantly higher compared to bright night sky. The results of this study will help solve the problems of the mental and physical effects of light pollution on astronomical observations and the reproduction of star images in planetariums.

## 1. Introduction

The stars are seen differently depending on the darkness of the sky, which may affect psychological feelings. In modern society, a phenomenon called “light pollution”, in which the stars in the night sky become less visible owing to increased artificial lighting, is particularly noticeable in urban areas [1]. Since ancient times, people have watched the stars, understood the movements of astronomical beings, and incorporated them into their lives and culture. However, as the starry sky becomes less visible, opportunities to feel connected to nature diminish, and people’s mental well-being can be affected [2]. Moreover, people living in areas susceptible to light pollution tend to perceive a lower naked-eye limit of magnitude as closer to the real starry sky [3], and an environment where children do not have a familiar view of the night sky may also reduce their opportunities to develop an interest in space and science. In an increasingly urbanized world, the simple experience of stargazing can be an important way to reaffirm the harmony between nature and people. These backgrounds require establishing stargazing preserves to protect the environment for stargazing and developing smart lighting technology to minimize light pollution [4], while the role of planetariums as facilities for enjoying the importance of the starry sky in public education is becoming increasingly important worldwide [5,6].

We have studied the reproduction and evaluation of star images in planetariums. As producing the same light as that of real stars in a planetarium is physically impossible, producing a perceptually equivalent appearance to that of real stars is important. In [7], three parameters of planetarium star images—“color”, “luminance”, and “size”—were adjusted, and the reproduction’s faithfulness was evaluated through psychophysical experiments. The results showed that the stars’ luminance and size had a significant effect on fidelity. In [8], the relationship between starry sky faithfulness (reproduction closer to the actual starry sky) and preferred starry sky (participants’ preference) was analyzed. Using star images of varying color, luminance, and size as visual stimuli, a psychophysical experiment with 47 participants revealed that men preferred reproductions with higher fidelity, while women preferred a more brilliant starry sky. Focusing on the reproduction of the Milky Way, [9] conducted a psychophysical experiment in which stars’ “brightness”, “size”, and “density” were adjusted and three evaluation indices—“faithfulness”, “preference”, and “depth feeling”—were used. The results showed that the density reproduction of the Milky Way was the most important factor, while the size of the stars had little effect on the ratings. To the best of our knowledge, this study is the first systematic investigation of reproduction methods that include visually complex elements such as the Milky Way and presents a new standard for starry sky representation in planetariums. These research results deepen the role that planetariums play in education and entertainment and demonstrate the importance of considering the visual characteristics of the participant and environmental factors. Additionally, by guiding the representation of the sky based on gender and personal preferences, they have provided a basis for improving night-sky display techniques and meeting diverse participants’ needs. Conversely, previous efforts have focused on the reproduction of star images and have not adequately considered the background of the star field, that is the night sky. The darkness of the night sky affects the human body and mind in several ways. For example, the brightness of the night sky and artificial light affect our circadian rhythms. In areas with high levels of light pollution, sleep quality tends to deteriorate, and circadian rhythms are disrupted [10]. As the darkness of the sky is an important piece of information for our mental and physical health, analyzing its relationship with human psycho-physiological states is important.

How can we determine the relationship between visual stimuli and psychophysiological states? Several studies have examined the effects of visual information in the natural environment on human psychophysiological states. For example, Song et al. showed that viewing images of forest environments produced more pronounced physiological and psychological relaxation effects than viewing images of urban environments [11]. Near-infrared spectroscopy (NIRS) measurements showed that viewing forest images produced relaxation effects, decreased hemoglobin and oxidized hemoglobin (OxyHb) concentrations, and improved comfort and naturalness scores in psychological ratings, which suggests that forest environments are beneficial for mental health. Moreover, viewing images of nature reduces orbitofrontal cortex (OFC) activity and improves mood [12]. Additionally, combined stimulation with forest-derived visual, auditory, and olfactory elements further enhances physiological and psychological relaxation [13]. Consequently, research endeavors are underway to quantify emotions and stress states through the measurement of biological information, particularly within medicine-related fields. NIRS has emerged as a particularly promising method for stress assessment and evaluation of relaxation-method efficacy [14]. Its efficacy in brain activity assessment related to mental stress has also been demonstrated [15]. Additionally, heart rate variability (HRV) has been proposed as a stress indicator and potential metric for assessing mental health [16]. Furthermore, HRV analysis of emotional responses to audiovisual stimuli was conducted, and HRV was measured in three genres of video: suspense, nature, and comedy [17]. This confirmed that HRV is a reliable tool for emotional response assessment. These findings suggest the potential for stress assessment through biological markers.

As reported in related studies [11], viewing natural scenes has been linked to a state of relaxation and mental and physical stability in many people and this may also apply to stargazing. However, the relationship between the darkness of the night sky and healing remains to be investigated. The present study aims to investigate this relationship by conducting psychophysiological experiments in a planetarium. The experiments use star images representing dark night sky (low light pollution environment) and bright night sky (high light pollution environment). Through these experiments, we seek to determine the appropriate darkness of the night sky for reproducing the starry sky in a planetarium, which is desirable for mental and physical well-being.

## 2. Methodology

This study aimed to elucidate the relationship between darkness and the healing of the night sky in planetariums. In addition to assessing psychological healing, we quantified the body’s physiological healing by measuring two types of physical stress states: OxyHb concentration in the OFC by NIRS, HRV, and movie stimuli (assuming that video images are viewed daily) and star image stimuli with different darkness in the night sky.

### 2.1. Experimental Stimuli

**Figure 1:**
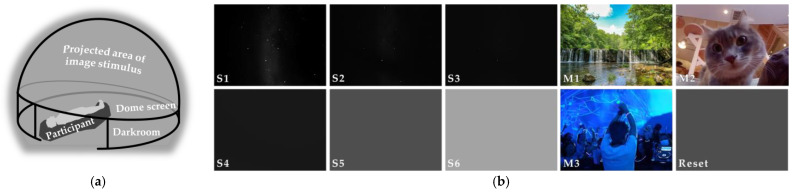
Experimental setup. (**a**) Experimental environment. (**b**) Experimental image stimuli. Note: Owing to the limitations of image tone on paper, the actual appearance is different.

We prepared 9 types of images as visual stimuli: 6-star images with different night sky darknesses and 3 non-star image movies for comparison, as summarized in Table 1. All were projected within a 3 m radius dome. All visual stimuli were presented to the participants’ full visual field for 180 s. This 180 s presentation time was designed to allow sufficient time for dark adaptation in total darkness, which requires 30 s or more. When switching stimuli, we presented the ambient light of 1 lx illuminance for 120 s as a reset stimulus. The experimental setup is illustrated in Figure 1.

- Star Image Stimuli:

The S1–S6 star image stimuli were all identical in terms of the projected brightness of the stars themselves and differed in the darkness of the stellar background field. The projected stellar field was a region of stars up to magnitude 6.5, centered on Orion. The stars were projected across the entire dome by a special planetarium projector installed inside the dome. The darkness of the night sky varied in tenfold increments of illuminance, from a dark starry sky imitating low light pollution to a bright starry sky imitating high light pollution. The star image stimulus was designed by measuring brightness inside the dome with an illuminance meter (CL-500A, Konica Minolta, Inc., Tokyo, Japan). Sky-quality meter (SQM) values, which measure the darkness of the night sky in magnitude per square arcsecond, were measured against the dome zenith using a device (SQM, Unihedron, Grimsby, Canada). For reference, the brightness of the city corresponding to the darkness of each night sky and the degree of moonlight are provided in Table 1. For example, a star image from S2 indicates that it corresponds to a starry sky in the light of a new moon or a starry sky observed on an isolated island with low light pollution. In general, when the field is dark, as in S1–S3 with SQM values of 20 or more, even the Milky Way is visible; when the field is bright, as in S6, which is close to the brightness of the sky 30 min after sunset, only a few high magnitude stars comprising the constellation Orion can be observed. To eliminate the influence of environmental noise inside and outside the experimental room, the sound of a rushing river in the forest was played during the presentation of the star image and reset stimuli. The dome is an air intake-type structure that maintains a hemispherical shape. Using natural sounds such as wind would amplify rather than cancel out the noise. For these reasons, a river sound stimulus was used to provide a stable and consistent sound flow while suppressing environmental noise. The same auditory stimuli are presented in the star image stimulus condition; thus, the only stimulus information that differs is the visual stimulus.

- Movie stimuli:

The star image stimuli S1–S6 were used to evaluate differences in the darkness of the night sky. However, we rarely experience such variations in night sky brightness in everyday life. Therefore, it is difficult to imagine the degree of healing that these comparison results may provide. To help readers understand the extent of the healing sensation indicated by the results, we conducted an experimental analysis using video stimuli M1–M3 for comparison. These audiovisual stimuli are more familiar and commonly encountered on a daily basis.

The M1–M3 movie stimuli included various video content, such as waterfall and cat content, which are nature and animal images that are expected to have a healing effect by inducing serotonin and oxytocin, and club videos, which are expected to cause unusual and intense stress. The movies were projected in the ambient light of 1 lx—the same illuminance as the reset stimuli. The movie stimuli were projected using a single-lens low-light projector (LSPX-P1, Sony Corp., Tokyo, Japan), and the maximum illumination in the images was controlled to 100 lx so as not to disturb the participants’ dark-adapted state. Auditory stimuli were attached to the movie and presented at a volume equivalent to that of the star image stimuli.

In this study, the loudness of the auditory stimulus was maintained at 30 dB. A study evaluating the effect of the sound level of a non-stress test (NST) device on stress parameters in women experiencing their first childbirth found that NST sound levels above 35 dB affected biological data, such as heart rate [18]. Therefore, the 30 dB sound level used in this study is considered to have had a minimal effect on biological data.

### 2.2. Experimental Procedure

To quantify the physical stress state induced by audiovisual stimuli, we performed two types of biometric measurements: the first was NIRS (Hb131S, Astem Co., Kanagawa, Japan), which non-invasively measures changes in OxyHb concentration in the OFC. The NIRS sensor was placed in the OFC region in a sun visor-like device, and blood OxyHb was measured at 0.5 s intervals from a total of four measurement channels—two channels each on the left and right sides—which allowed us to assess changes in brain activity in response to visual stimuli. The second was a heart rate monitor (WHS-1, Union tool Co., Tokyo, Japan) to measure HRV. This wearable heart rate sensor was placed on the participant’s solar plexus and measures heart rate and body movement at 1 s intervals. HRV was measured to assess parasympathetic and sympathetic responses, and autonomic nervous system activity was also monitored.

Participants were first fitted with heart rate and NIRS measurement devices. After adapting to the environment, including lighting and room temperature, participants lay down on a cushioned sofa in a comfortable position while the reset stimuli were presented and the experiment was explained to them. Measurement devices continuously measured during the experiment. We also asked the participants to view the stimuli by switching their eyes without moving their bodies as much as possible during stimulus observation to avoid influences on the biological information. After viewing the randomly presented audiovisual stimuli, participants were switched to the reset stimuli. They then responded to the degree of healing on an 11-point Likert scale (0–10), with 0 representing no healing at all and high stress and 10 representing the highest degree of healing. The reset stimulus was presented for 120 s, after which the experimenter moved on to the next stimulus presentation.

To minimize the influence of variations in physical data owing to body condition, we took measurements three times per participant and used the average as measured data. Additionally, to avoid the influence of memory for the previous assessment, we performed each assessment at least one week later than the previous one and presented stimuli in a random order.

## 3. Results

To minimize individual differences in gender, age, and body size, we included five healthy young Japanese women (mean age 26.4 ± 4.6 years) in this experiment as participants. Since significant changes in HRV by gender and age have been confirmed [19], this study targeted young women who frequently visit planetariums. Note that all participants were naive, having no experience with daily astronomical observations or viewing star images in planetariums. They were born and raised in Japan, where the night sky corresponds to the dark night sky conditions of the S3 and S4 stimuli. The study was conducted according to ethical guidelines.

### 3.1. Physiological Measurements

The biometric data of OxyHb and the heart rate from NIRS measurements of OFC were analyzed for each time interval after noise-reduction preprocessing. According to a reported study analyzing NIRS data at a stimulus presentation time of 30 s, the maximum change in OxyHb concentration occurred within the first 10 s [20]. Therefore, in this study, to observe the trend over time, we analyzed the mean value during the 10 s immediately after stimulus presentation as the short time change (hereafter referred to as “short”), the mean value during the 30 s after stimulus presentation as the medium time change (“mid”), and the mean value during the 180 s of total stimulus presentation as the long time change (“long”).

#### 3.1.1. OxyHb in OFC

OxyHb in the OFC, measured in two channels each on the left and right side, was obtained as the average OxyHb change in the left and right prefrontal cortices, respectively. The left prefrontal cortex tends to be more active during relaxed positive emotional stimuli, while the right prefrontal cortex tends to be more active during negative emotional stimuli under stress. Here, we analyze this using the asymmetry index (AI), which quantifies the relative balance of left and right activity; the AI index is commonly used in NIRS and EEG (electroencephalography) studies in particular and has been widely applied in studies of emotion, stress, cognition, and psychiatric disorders [21]. This index can be used to evaluate differences in the balance between left and right brain activity and to visualize and analyze subtle differences. AI is calculated by the following equation, which consists of the ratio of left and right OxyHb changes (Δ):

$$AI=({\Delta OxyHb}_{left}-{\Delta OxyHb}_{right})/\;({\Delta OxyHb}_{left}+{\Delta OxyHb}_{right})$$

AI values generally range from −1 to 1, with AI > 0 indicating that the left prefrontal cortex is more active, AI < 0 indicating that the right prefrontal cortex is more active than the other, and AI = 0 indicating that the left and right sides are equally active. Moreover, the right side is more active in chronic stress conditions such as anxiety [22], and the left side is more active in cognitive tasks such as language tasks [23].

**Figure 2:**
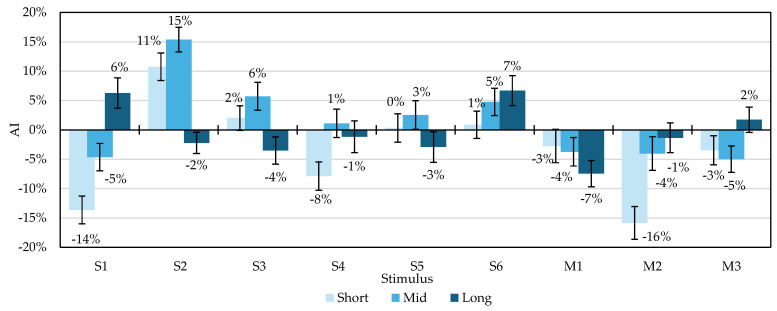
Asymmetry index for each stimulus.

The analysis of the AI for each time section is illustrated in Figure 2, where the mean index value of the AI is shown as a percentage and each standard error is shown as an error bar. The trends in AI for each period are as follows:

- Short (10 s): In general, the tendency for the AI is to change significantly with the stimulus. In particular, M2 (−16%), S1 (−14%), and S4 (−8%) show bias toward the right prefrontal cortex, indicating that the participants were under stress. Conversely, S2 shows positive bias toward the left prefrontal cortex (+11%), indicating that a relaxed positive state was reached immediately after the stimulus presentation.
- Mid (30 s): The changes in AI are milder than in short, but S2 (+15%) shows bias toward the left prefrontal cortex.
- Long (180 s): Compared to the short and mid periods, the long period shows less bias between stimuli owing to the long period averaged over a longer period, but the long period shows a difference in the overall balance of left and right brain activity during the stimulus presentation. Interesting trends can be observed that were not observed in the short and mid-time periods. For example, the right prefrontal cortex was dominant in the short and mid period, but the left prefrontal cortex was dominant overall in S1. This indicates that the participants, who were stressed by the pitch darkness soon after the stimulus presentation, were able to feel healing when they saw the Milky Way and other starry sky images as their dark adaptation accelerated. Conversely, some stimuli, such as S2 and S3, were dominant in the left prefrontal cortex in the short and mid period but were dominant in the right prefrontal cortex overall. The reason for this might be that the participants felt healing immediately after the stimulus presentation but became bored with the stimuli because visibility did not change, and the stimuli were monotonous even after dark adaptation was accelerated.
- The trends in AI for each stimulus type are summarized below:
- Star image stimuli S1–S6: Short time shows strong bias, particularly in S1 (−14%) and S2 (+11%). Mid-time shows a strong trend in S2 (+15%), but the other stimuli are relatively stable. S1 and S6 are slightly biased toward the left prefrontal cortex in the overall long period.
- Movie stimuli M1–M3: M2 shows −16% bias toward the right prefrontal cortex in the short period, and M3 was biased toward the left prefrontal cortex, as was S1, in the short period.
- In summary, specific stimuli (e.g., S1, S2, and M2) caused substantial changes in the characteristics of each time interval. Overall, the variation of AI for each stimulus was larger in the order of short > mid > long; this confirms that the shorter the stimulus presentation time, the more actively the left and right prefrontal cortices responded.

**Figure 3:**
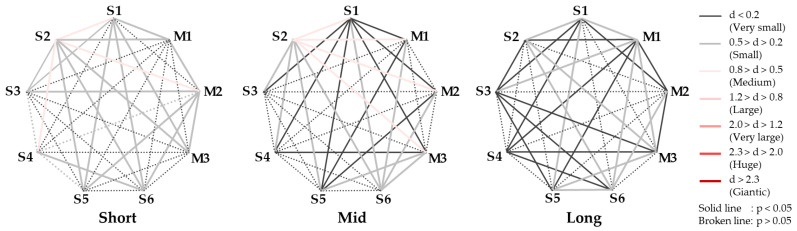
The *p*-value and effect size in AI for each stimulus pair.

Next, *p*-values and effect sizes (d) were calculated in the corresponding *t*-tests for the AI for each stimulus obtained to verify the significance of the relaxation state owing to the balance of left and right brain activity. The *p*-values and effect sizes for each pair of comparison stimuli are depicted in Figure 3. In this study, the effectiveness criterion for effect size d is based on Cohen and Sawilowsky [24,25]. The effect size refers to “the degree of difference to be detected” and is an indicator of the experiment’s effect. The *p*-value and effect size of a statistical test provide different information: a low *p*-value indicates a statistically significant difference but does not indicate how large the difference is. Conversely, effect sizes are useful in interpreting the substantive meaning of a study because they indicate the magnitude of the difference and are independent of the sample size. Therefore, when considering the results of a statistical test, considering both the *p*-value and effect size is important. For the sample size of participants in this experiment, effect sizes greater than ‘large’ are considered to be reliable significant differences. In this study, we discuss the *p*-values of *p* < 0.05 and effect size *d* > 0.5 (medium), including the possibility of reaching significance.

In the comparison pairs of S2-{S1, S4, M2} in short and S2-{M1, M2, M3} in mid, the *p*-values were significant and the effect sizes larger than medium. These results indicate that S2 significantly activated the left prefrontal cortex more than the other stimuli and that the darkness of the night sky in S2, which is known as the darkness of the beautiful starry sky with low light-pollution effects that can be seen from the Earth, is also a relaxing and positive state from the viewpoint of brain activity. S1, which was significantly more dominant in the right prefrontal cortex immediately after the stimulus presentation compared to S2, showed a shift to a dominant left prefrontal cortex as time passed. This suggests that when one adapts to darkness, star-image stimulation has a healing effect that is sufficient to alleviate the stress caused by darkness.

#### 3.1.2. Heart Rate Variability

**Figure 4:**
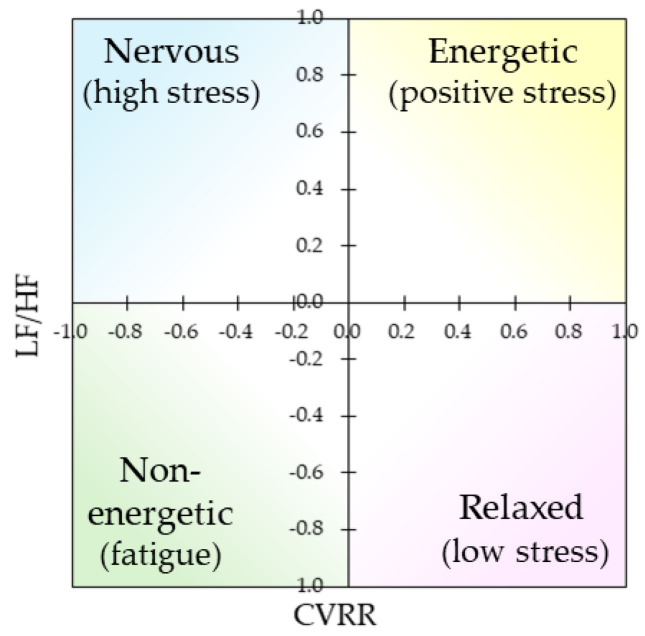
Relationship between CVRR and LF/HF indices and relaxation state.

For the measured HRV, CVRR and LF/HF indices were calculated for each time period. CVRR is one of the indices of HRV and reflects parasympathetic activity, with a high value indicating a relaxed state and a low value indicating a stressed state. Furthermore, LF/HF is an index of the balance between the sympathetic and parasympathetic nervous systems calculated from HRV, with higher values indicating sympathetic dominance and lower values indicating parasympathetic dominance. By obtaining both z-scores, the state of autonomic function can be visualized relative to each other, as shown in Figure 4. Sympathetic dominance in the first quadrant (top right: high CVRR and high LF/HF) with high HRV indicates a state of stress and an active sympathetic nervous system. It is seen during exercise, excitement, or in people with high stress tolerance, and is in a state of energetic and positive stress. In the second quadrant (upper left: low CVRR and high LF/HF), when the sympathetic nervous system is dominant and HRV is low, the sympathetic nervous system is overactive and the parasympathetic nervous system suppressed owing to a high stress load, tension, or overwork, which may indicate chronic stress or an autonomic nervous disorder. In the third quadrant (lower left: low CVRR and low LF/HF), when the parasympathetic nervous system is dominant and HRV is low, the autonomic nervous system is in an overall state of reduced activity. Tiredness, poor physical condition, or decreased autonomic function is observed, possibly owing to low energy from exhaustion or overexertion. The fourth quadrant (lower right: high CVRR and low LF/HF), parasympathetic dominance and high HRV, is observed in a relaxed state or during recovery or rest. During sleep, meditation, and deep relaxation, sympathetic activity is suppressed, and parasympathetic dominance emerges. Thus, analyzing the relationship between CVRR and LF/HF in each quadrant allows us to assess the balance of the autonomic nervous system and state of stress.

**Figure 5:**
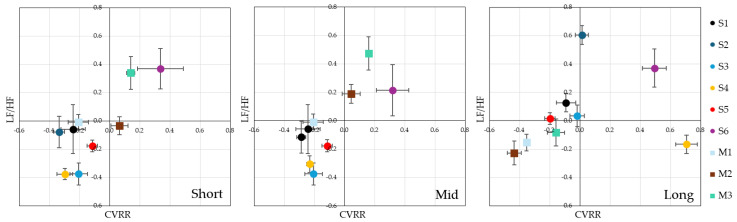
Average z-score of CVRR, LF/HF for each stimulus (from **left** to **right**: short, mid, long).

The results of the analysis of CVRR and LF/HF for each time interval are illustrated in Figure 5. Each value in the bar graph is a Z-score, and each standard error is indicated by an error bar. The scatterplots also show CVRR on the horizontal axis and LF/HF on the vertical axis, summarizing the state of autonomic function in each stimulus and time interval condition so that they can be compared, as in Figure 4.

Various effects are observed, such as an increase in CVRR and an increase or decrease in LF/HF depending on the stimulus. For example, S6 has a high LF/HF and may increase sympathetic activity. Conversely, M1 has a high CVRR and low LF/HF, which can lead to parasympathetic dominance.

Autonomic balance may change over time (short to mid to long). Different autonomic responses are observed for each stimulus, which suggests different effects of sympathetic and parasympathetic nerves.

The time course of each stimulus is summarized as follows:

- S1: CVRR increased slightly with a longer observation time.
- S2: LF/HF increased significantly in the long period, and sympathetic activity tended to increase.
- S3: Both CVRR and LF/HF were small in the short and mid periods, but both took values near zero in the long period, which indicates a transition to a neutral state.
- S4: Both CVRR and LF/HF were small in the short and mid periods, but CVRR increased significantly in the long period, which suggests parasympathetic activity.
- S5: A slight tendency toward sympathetic dominance was observed in the long period, but the change was small.
- S6: LF/HF was consistently high, probably owing to the sympathetic dominance of the stimulus.
- M1: CVRR remained consistently slightly lower, and LF/HF tended to decrease over time, indicating a gradual shift toward parasympathetic dominance.
- M2: The sympathetic nervous system was temporarily activated in the mid period and decreased in the long period. Stress might have been temporarily accelerated by the cat video content.
- M3: CVRR did not change in the short and mid periods but decreased in the long period. LF/HF was high in the short and mid periods but decreased to around 0 in the long period. A long period may decrease CVRR and increase sympathetic nerve activity.

Overall, the direction of change in CVRR varied by stimulus, with some stimuli increasing toward the long period (S2, S4), some decreasing (M2, M3), and some consistently high (S6). For LF/HF, some stimuli, such as S6, had consistently high LF/HF and stress (sympathetic dominance).

**Figure 6:**
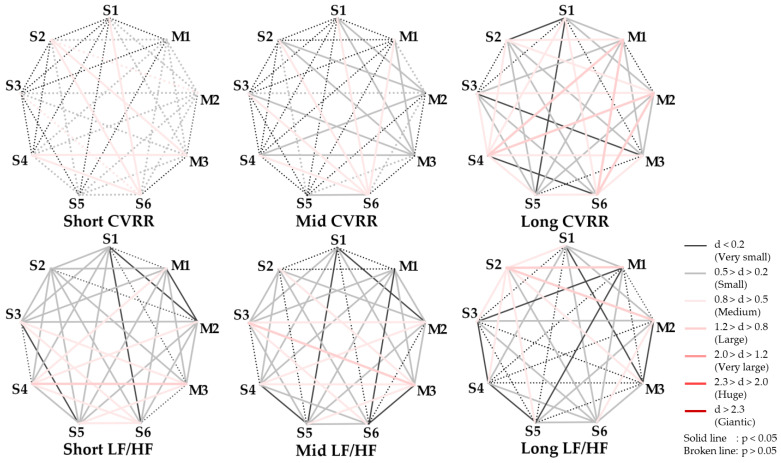
The *p*-value and effect size in CVRR and LF/HF for each stimulus pair.

We then tested the significance of the relaxation state between each stimulus for the CVRR and LF/HF indices for each of the above stimuli by calculating the *p*-values and effect sizes in the corresponding *t*-tests. The results are presented in Figure 6. As in Figure 3, the comparison stimulus pairs with significant differences are shown in index, together with the numerical values of the *p*-values and effect sizes: comparing S4-{S6, M3} in the short period, S3-S6 in the mid period, and S2-{S4, M1, M2,} and S6-M2 in the long period, we found that both CVRR and LF/HF had significant *p*-values and effect sizes greater than medium, indicating strong differences. Overall, S2 activated the left prefrontal cortex significantly more than the other stimuli, and the darker night sky of S2, known as the darkness of a beautiful starry sky with less light pollution visible from the Earth, was a relaxed and positive state in terms of brain activity.

These results suggest that S6, a bright night sky immediately after sunset, is brighter than other stimuli and that the sympathetic nervous system is more physically active, resulting in an energetic and positive state of stress. This makes sense because it is consistent with the physiological phenomenon of the body suppressing activity toward sleep as time passes after sunset. Conversely, when we focused on the dark night sky, we found that CVRR and LF/HF were significantly lower with the dark night sky than with other stimuli over time, as in S4, S3, and S2 for the short, mid, and long periods, respectively, indicating a change in the trend toward reduced autonomic nervous system function. This suggests that dark adaptation accelerates over time and that the body is changing toward lower energy.

### 3.2. Psychological Assessment

**Figure 7:**
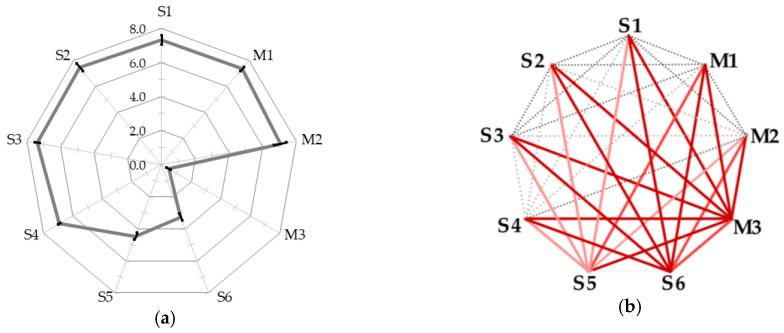
Average rated value and significant difference. (**a**) Average rated value of psychological healing for each stimulus with error bars of standard errors. (**b**) The *p*-value and effect size for each stimulus pair.

Participants rated the degree of “healing” on an 11-point Likert scale after viewing each stimulus; the average rating of psychological healing by five participants, with three ratings per participant, is shown in Figure 7a. A higher level of healing is observed for the star images S1–S2 in the darker night sky, and the rating decreases as the night sky becomes brighter. For the movie stimuli, the psychological rating was higher for M1 and M2, in that order, and M3 was almost non-healing.

We tested significance by calculating *p*-values and effect sizes in the corresponding *t*-tests to verify the significance of the psychological healing level between each stimulus for the mean rating in each stimulus. The results are presented in Figure 7b. The resulting stimulus pairs that were valid for both the *p*-value and effect size were {S5, S6}-{S1, S2, S3, S4, M1, M2, M3} and M3-{all}, which indicates that stimuli S5, S6, and M3 were significantly less healing than the others.

### 3.3. Comprehensive Discussion

**Figure 8:**
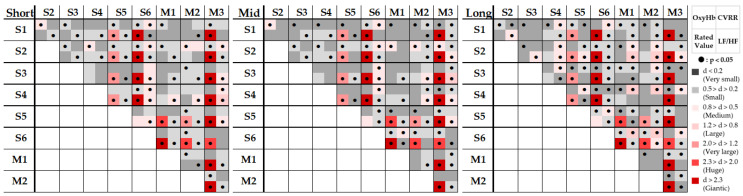
Significance maps.

The results of the physiological measurements and psychological assessment of healing reported in this chapter are summarized in a comprehensive summary. The significance of both *p*-values and effect sizes (medium and above) between each stimulus in each physiological–psychological measure was simply counted and is shown as significance maps in Figure 8.

First, we compared the star images. We confirmed that S6, a bright night sky shortly after sunset, was significantly less mentally healing and more physically positive than the other darker night sky. In short observation, S1 (low healing)–S2 (high healing) changed to S1 (high healing)–S2 (low healing) over time. S1–S3 were not significant regardless of time. With the movie stimuli, M3 tended to be significantly more stressed—both psychologically and physiologically—than with the other stimuli. Additionally, the results of each measurement suggest that the superior relationship of healing by physiological and psychological evaluations may not be equivalent; however, as external factors other than stimuli can be received as noise in biological measurements, psychological evaluation is still considered more reliable.

Table 2 presents the correlations between psychological assessment scores and each physiological measurement index (colored cells indicate significance at *p* < 0.05). The results of significant correlations confirmed that CVRR and LF/HF, and the left–right ratio of OxyHb concentration in OFC were correlated with psychometric values immediately after stimulus presentation up to 30 s and for a long time, respectively. The physiological measures that became valid changed with time, which indicates the subtlety of physical information owing to the effects of fatigue and habituation over time. Illuminance, which indicates physical brightness, also showed a significant correlation with psychometric scores. These results suggest that the human body is affected by the healing/stress of physical illuminance, which indicates the darkness of the night sky and that the physical state changes moment by moment, depending on the elapsed time exposed to the environment. Moreover, HRV for a short time and OxyHb concentration for a long time are related to psychological state.

In addition, the sound level used in this study was kept at 30 dB, which would have had minimal effect on the biological data. However, we cannot rule out the possibility that the physiological data were affected by differences in auditory stimuli, such as melodies, between the starry sky and movie stimuli. As a future topic, analysis down to the sound level should also be considered. Furthermore, in this study, as is common in psychophysical approaches, age, gender, and familiarity with astronomical observations were kept equal, and the analysis was conducted under conditions that minimized the diversity of participants in the experiment as much as possible. However, since taking diversity into account may allow for a richer analysis and discussion that can be applied to all humans, taking these factors into account is also a future topic.

## 4. Conclusions

In this study, we constructed an experimental environment that mimics a dark night sky (low light pollution environment) and a bright night sky (high light pollution environment) using star image stimuli in a planetarium, and we analyzed the relationship between the darkness of the night sky and the state of human physical and mental healing through psychophysiological experiments. In addition to the evaluation of psychological healing, we evaluated physiological healing of the body by quantifying the state of physical stress by measuring OxyHb concentration in OFC by NIRS as well as HRV. In addition to star image stimuli with different night sky darkness, movie stimuli were also used as experimental stimuli, assuming that the participants watched movies daily. Five healthy young Japanese women participated, and the measured data were statistically analyzed.

The results of the experimental analysis showed that the appropriate night sky darkness in a planetarium’s reproduction of the night sky, which is desirable for the human mind and body, is the 10-3 darkest night sky observable on Earth, which can be seen in areas with almost no light pollution and under new moon conditions. Additionally, participants felt stressed immediately after viewing the stimulus for the even darker 0 lx night sky because it was excessively dark, but over time, they changed to feeling healing owing to the promotion of adaptation to darkness. The results of HRV and psychological assessment showed a significant correlation for a short time—approximately 30 s after stimulus presentation—confirming that LF/HF can be a valid indicator. Moreover, illuminance, which indicates physical brightness, and psychological rating also showed a significant correlation, which indicates that the information of darkness in the night sky affects the degree of healing.

The approach of this study suggests that the environmental factor of night sky darkness may have an impact on mental health. Light-pollution control efforts to protect the darkness of the night sky and the use of star images in planetariums for relaxation may improve quality of life. Due to the limited experimental conditions in this study, making policy recommendations remains challenging. However, the findings indicate that dark urban lighting, free from light pollution, is desirable from a healing perspective. Additionally, the results suggest that the ideal night sky in planetariums would be better to replicate the darkness of a new moon, which is not easy to achieve in many planetarium facilities and astronomy education programs.

In future studies, we plan to expand the sample size and include participants with varying demographic backgrounds to further validate and generalize our findings. However, for this initial study, we believe our approach is appropriate for the research objectives. In addition, the experimental stimuli used, which included star images and movie clips, might not fully capture the range of visual experiences encountered in daily life. To address this, future research could benefit from incorporating a more diverse set of stimuli, such as dynamic natural or urban scenes, to provide a more comprehensive assessment of how different visual stimuli affect psychological and physiological states.

## Figures and Tables

**Table 1:** Experimental stimuli.

Stimulus	Image Stimuli	Auditory Stimuli	Illuminance [lx]	SQM (Representative Place in Light Pollution Map [1])
S1	Perfect dark starry sky	The sound of a rushing river in the forest	0	>23 (Universe)
S2	Starry sky lit by the new moon		0.001	21.94 (Isolated islands, mountains, wildlands)
S3	Starry sky lit by the crescent moon		0.01	20.07 (Suburbs)
S4	Starry sky lit by the full moon		0.1	16.87 (Large cities, downtown)
S5	Starry sky lit by street lamps		1	14.43
S6	Sky 30 min after sunset		3	13.36
Reset	Ambient light without star image		1	-
M1	Waterfalls		1	-
M2	Cat		1	-
M3	Club		1	-

**Table 2:** Correlation between psychological assessment scores and each physiological measurement index.

	OxyHb (AI)	CVRR (z-Score)	LF/HF (z-Score)	Illuminance [lx]
Short	−0.11	−0.72	−0.59	−0.57
Mid	0.22	−0.77	−0.71	
Long	−0.57	−0.08	0.01	

The colored cells indicate significant differences at *p* < 0.05.

## Data Availability

Data are available on request owing to restrictions.
